# Antiangiogenic, Anti-Inflammatory and Antioxidant Properties of *Bidens tripartite* Herb, *Galium verum* Herb and *Rumex hydrolapathum* Root

**DOI:** 10.3390/molecules28134966

**Published:** 2023-06-24

**Authors:** Katarzyna Antoniak, Elżbieta Studzińska-Sroka, Marcin Szymański, Marlena Dudek-Makuch, Judyta Cielecka-Piontek, Katarzyna Korybalska

**Affiliations:** 1 Independent Researcher, 92-507 Łódź, Poland; antoniakkatarzyna@wp.pl; 2 Deparatment of Pharmacognosy and Biomaterials, Poznan University of Medical Science, Rokietnicka 3 Str., 60-806 Poznań, Poland; jpiontek@ump.edu.pl; 3 Centre for Advanced Technologies, Adam Mickiewicz University in Poznań, Uniwersytetu Poznańskiego 10 Str., 61-614 Poznań, Poland; marcin.szymanski@amu.edu.pl; 4 Regulatory Affairs Department, Curtis Health Caps S.A., Batorowska 52 Str., Wysogotowo, 62-081 Przeźmierowo, Poland; mdudek-makuch@chc.com.pl; 5 Department of Patophysiology, Poznań University of Medical Science, Rokietnicka 8 Str., 60-806 Poznań, Poland; koryb@ump.edu.pl

**Keywords:** polyphenols, free radicals, invasiveness, migration, proliferation

## Abstract

Plants are commonly used in folk medicine. Research indicates that the mechanisms of biological activity of plant extracts may be essential in the treatment of various diseases. In this respect, we decided to test the ethanolic extracts of *Bidens tripartita* herb (BTH), *Galium verum* herb (GVH), and *Rumicis hydrolapathum* root (RHR) on angiogenic, anti-inflammatory, and antioxidant properties and their total polyphenols content. In vitro studies using endothelial cells were used to see tested extracts’ angiogenic/angiostatic and anti-inflammatory properties. The DPPH assay and FRAP analysis were used to detect antioxidant properties of extracts. The Folin-Ciocalteu analysis was used to determine the content of total polyphenols. The results of gas chromatography–mass spectrometry analysis was also presented. In vitro study demonstrated that BTH, GVH, and RHR ethanolic extracts significantly increased cell invasiveness, compared with the control group. Increased endothelial proangiogenic invasiveness was accompanied by reduced metalloproteinase inhibitor 1 (TIMP-1) and raised in metalloproteinase 9 (MMP-9). Only BTH and GVH significantly reduced cell proliferation, while BTH and RHR facilitated migration. Additionally, tested extracts reduced the production of proangiogenic platelet-derived growth factor (PDGF) and hepatocyte growth factor (HGF). The most potent anti-inflammatory capacity showed BTH and GVH, reducing proinflammatory interleukin 8 (CXCL8) and interleukin 6 (Il-6), compared to RHR extract that has slightly less inhibited CXCL8 production without affecting IL-6 production. Moreover, we confirmed the antioxidant properties of all examined extracts. The highest activity was characterized by RHR, which has been correlated with the high content of polyphenols. In conclusion, the modifying influence of examined extracts can be promising in disorders with pathogenesis related to angiogenesis, inflammation and free radicals formation. BTH is the best choice among the three tested extracts with its antiangiogenic and anti-inflammatory properties.

## 1. Introduction

Plants are a source of active compounds with multidirectional biological activity. Extracts from various parts of plants are used in traditional medicine and conventional medicine to support the therapy of multiple diseases. Therefore, plant preparations (e.g., extracts, tinctures, juices) can also be used for conditions caused by an imbalance between pro- and antiangiogenic milieu pro-, and antioxidant, pro and anti-inflammatory factors [1].

Angiogenesis occurs during organ development and the postnatal stage. New vessel formation is expected during the wound-healing and may contribute to the progression of disorders such as cancer, psoriasis, retinopathies, and other angiogenic diseases [2]. Angiogenesis is a multistep process, including the interaction between cells, extracellular matrix (ECM), and angiogenic/angiostatic factors, where endothelium plays a leading role. The proliferation, migration, and endothelial cell invasiveness depend on the surrounding ECM and remain under the control of the growth factors (angiogenic factors), metalloproteinases (MMPs), and their inhibitors (TIMPs). The balance between angiogenic and angiostatic factors promotes the sprouting of new capillaries [2].

Free radicals created by hypoxia, hypoxia-reoxygenation, and during inflammation initiate oxidative stress. Radicals themselves and their peroxidized metabolites may act as proangiogenic factors. They lead to wound healing with concomitantly new blood vessel formation [2,3].

*Bidens tripartita* herb (BTH), *Galium verum* herb (GVH), and *Rumex hydrolapathum* root (RHR) are medicinal raw materials used for a long time in the traditional medicine of various nations.

*Bidens tripartite* L. (Bur-marigold), *Asteraceae*, occurs commonly in humid regions, mainly in the Northern hemisphere. In folk medicine, the aerial parts of *B. tripartite* are used in the treatment of catarrhal rhinitis, fevers, colds, bladder and kidney complaints, in enteritis, diarrhea, and colitis, as well as orally and externally in the treatment of skin diseases, acne, eczema, psoriasis [4,5]. The phytochemical studies shown *B. tripartite* seconday metabolites are flavonoids, phenolic acids, tridecane-derived polyacetylenes, hydroxycoumarins, sterols, essential oil, and flavonoids [4,6] and the presence of these compounds can justify their utilization in traditional treatment. The scientifical study shown that *B. tripartite* exhibit the biological activity. Previous in vivo experiments demonstrated its antiulcer and anti-inflammatory activities [4]. Moreover pharmacological studies revealed that the *B. tripartita* possesses antioxidative [5,6,7], antimicrobial, cytotoxic and antiproliferative properties [6].

*Galium verum* L. (Lady’s Bedstraw) *Rubiaceae*, occuring widely in a temperate climate. The literature data shown this plant has been traditionally used in the treatment of skin disorders, exogenous treatment of psoriasis, and delayed wound healing. It was also used in tongue cancer, cancer ulcers, and breast cancer, furthermore it recommended for the therapy of cystitis and rheumatic diseases [8,9,10]. The phytochemical studies of the species revealed the presence of iridoid glycosides, flavonoids, phenolic acids, anthraquinones, monoterpene glycosides, phytosterols, and essential oil [8,11]. So far, experimental studies proved that the extract of *G. verum* is characterized by antioxidant activities in various models [12,13,14] and also possesses antimicrobial [8], and immunomodulatory activities [15].

One of the widely distributed in Europe and North America species of Polygonaceae family is *Rumex hydrolapathum* Huds. In traditional medicine, *R. hydrolapathum* has been used in diarrhea, constipation, also in skin diseases, such as eczema, rashes, and wounds [16]. The phytochemical analysis shown the roots of *Rumex* species are rich in biologically essential substances, such as anthraquinones: mainly emodin, chrysophanol, and physcion and its derivatives [17], tannins, naphthalene derivatives (nepodin, nepodin-8-*O*-β-glucoside), benzofuranones, sterols, ascorbic acid, oxalic acid, and other minerals [16]. The different studied demonstrate the interesting biological potential of *Rhumex* sp. The extracts from roots of different *Rumex* species exerted high antibacterial activity [18]. Pharmacological studies conducted on the radix of *Rumex* spp. indicate its anti-inflammatory [19,20], analgesic, antipyretic, antioxidant [16,20,21,22,23], and also anti-cancer activities [23]. Studies also confirm once-used applications in skin disease. *R. crispus* and its active component, chrysophanol may affect the course of allergic diseases such as atopic dermatitis [19,24].

In this context, our study examines the antioxidant properties of BTH, GVH, and RHR and their ability to modulate angiogenesis, inflammation, and oxidative stress accompanying the pathomechanism of various diseases.

## 2. Results

### 2.1. GC-MS Analysis of the Extracts

The volatile compounds of BTH, GVH, and RHR alcoholic extracts were analyzed using the GC–MS and the qualitative and quantitative compositions are presented in Table 1. The chromatograms are presented in Supplementary Materials (Figures S1–S3). On the basis of the obtained results, 13, 5, and 14 different compounds were identified in BTH, GVH, and RHR extracts, respectively. The major components were Z,Z)-9,12-octadecadienoic acid (29.60%) and 1,2,3,4-tetrahydro-3-O-methyl-papaveroline (22.14%) in BTH, 7-ethyl-4-decen-6-one (45.95%) and 4-hydroxy-benzenepropanoic acid (41.40%) in GVH as well as 1,8-dihydroxy-3-methyl-9,10-anthracenedione (syn. chrysophanol) (32.04%) and 4-methyl-4-hepten-3-one (22.73%) in RHR. The presence of some compound was also detected by other authors. Chrysophanol in RHR was described by Demirezer et al. [25]. Moreover, hydroxycinnamic acid derivatives were recorded in species of the genus *Galium* sp. [26]. Fatty acids were also detected in BTH. Similarly to our results, Oproshanskaya detects palmitic acid in BTH extract [27].

### 2.2. Antioxidant Activity and Polyphenolic Content

As we demonstrated, all analyzed herbal extracts exerted antioxidant activity. RHR has the most potent antioxidant properties among the examined extracts. For GVH and BTH, this effect was about ten and about twenty times weaker than for RHR, respectively using the DPPH assay. The FRAP test also confirmed the highest antioxidant activity of the RHR extract. Moreover, antioxidant potential was only five times lower than the potent antioxidant vitamin C. All numeric values are presented in Table 2. The antioxidant activity correlated with the polyphenols content detected in these plants in high contents. RHR, containing five and ten times more polyphenols than GVH and BTH herbs.

### 2.3. Effect of BH, GVH, and RR on Endothelial Cells Viability

**Figure 1:**
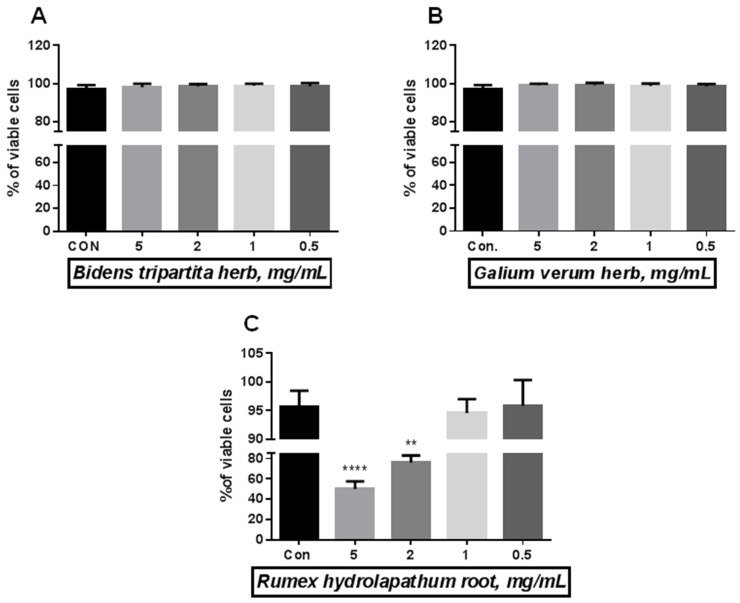
The effect of *Bidens tripartite* herb (BTH), *Galium verum* herb (GVH), and *Rumex hydrolapathum* root (RHR) on the endothelial cell viability Endothelial cells were exposed for 24 h to the control medium, and medium supplemented with herbal extracts at increasing concentration. The data were interpreted with repeated measures analysis of variance–One-way ANOVA, using a post hoc test for multiple comparisons (Dunn’s or Tukey’s tests). The results are expressed as mean ± SD, derived from three independent experiments. The data was expressed as percent of viable cells. Statistical significance: ** *p* < 0.01, **** *p* < 0.0001.

Using the Trypan blue exclusion test, we tested increasing concentration of herbal extracts from 0.5 mg/mL to 5.0 mg/mL. The preliminary viability test evaluates the highest level of tested extracts that is safe for cells (Figure 1A–C). For further analysis, we selected the following concentrations 5 mg/mL for the BTH (Figure 1A), 5 mg/mL for the GVH (Figure 1B), and 1 mg/mL for the RHR (Figure 1C).

### 2.4. Cell Proliferation, Migration and Invasiveness

**Figure 2:**
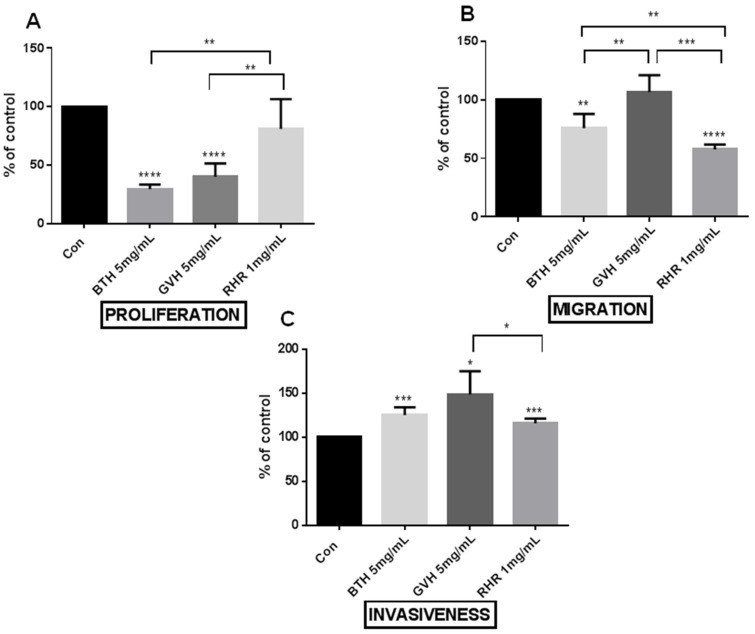
The effect of *Bidens tripartite* herb (BTH), *Galium verum* herb (GVH), and *Rumex hydrolapathum* root (RHR) on the endothelial cell proliferation (**A**), migration (**B**) and invasiveness (**C**). Endothelial cells were exposed for 24 h to the control medium, and medium supplemented with herbal extracts at the highest concentration that does not change cell viability. The data were interpreted with repeated measures analysis of variance–One-way ANOVA, using a post hoc test for multiple comparisons (Dunn’s or Tukey’s tests). The results are expressed as mean ± SD, derived from three independent experiments. The data was expressed as percent of control, where control (control = 100%). Statistical significance: * *p* < 0.05, ** *p* < 0.01, *** *p* < 0.001, **** *p* < 0.0001.

BTH significantly reduces cell proliferation (29%), migration (76%), and increases cell invasiveness by 25% when compared with the control group (Figure 2A–C). GVH and RHR also affect angiogenesis but less pronounced. Both extracts increase cell invasiveness, GVH by 49%, and RHR by 16% (Figure 2C). GVH, similarly to BTH, reduces cell proliferation (40%) but does not change cell migration (107%) (Figure 2A,B), which decreases RHR (58%) (Figure 2B).

### 2.5. Production of Angiogenic/Angiostatic Factors

**Figure 3:**
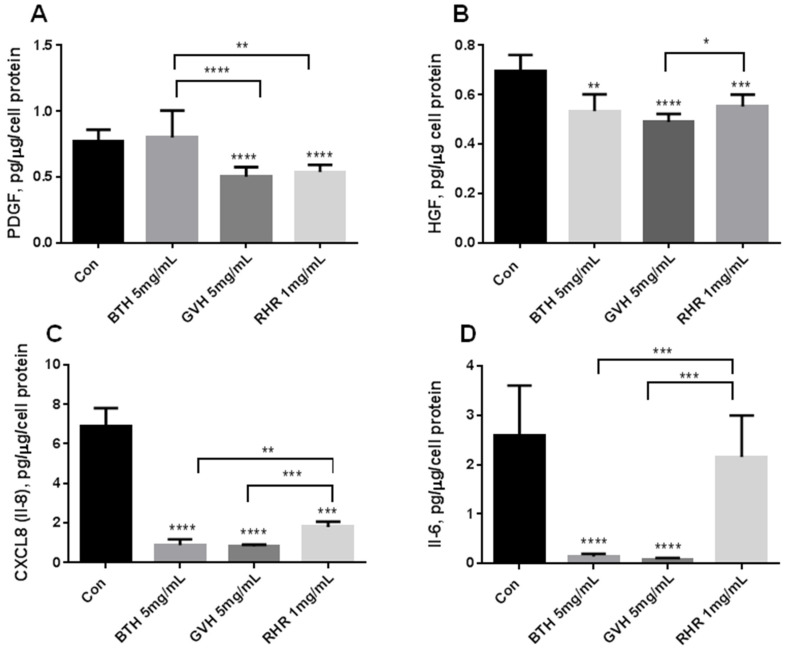
The effect of *Bidens tripartite* herb (BTH), *Galium verum* herb (GVH), and *Rumex hydrolapathum* root (RHR) on the endothelial cell production of angiogenic mediators released in the culture medium: PDGF (**A**); HGF (**B**); CXCL8 (**C**); Il-6 (**D**). Endothelial cells were exposed for 24 h to the control medium, and medium supplemented with herbal extracts at the highest concentration that does not change cell viability. The data were interpreted with repeated measures analysis of variance–One-way ANOVA, using a post hoc test for multiple comparisons (Dunn’s or Tukey’s tests). The results are expressed as mean ± SD, derived from three independent experiments, and were calculated per µg of cell protein. Abbreviations: PDGF- platelet-derived growth factor, HGF–hepatocyte growth factor, IL-8–interleukin 8, Il-6–interleukin 6. Statistical significance: * *p* < 0.05, ** *p* < 0.01, *** *p* < 0.001, **** *p* < 0.0001.

GVH reduces PDGF and HGF by about 30% (Figure 3A,B). BTH reduces only HGF production by 23% and does not change PDGF level. Both extracts vigorously reduce pro-inflammatory cytokines when compared to the control cells (IL-8: BTH and GVH by about 88%; IL-6: BTH and GVH by about 95%) (Figure 3C,D). RHR reduces PDGF and HGF by 30% and 20%, respectively (Figure 3A,B). RHR only reduced the production of the CXCL8 chemokine by 74% and did not change IL-6 production (Figure 3C,D).

**Figure 4:**
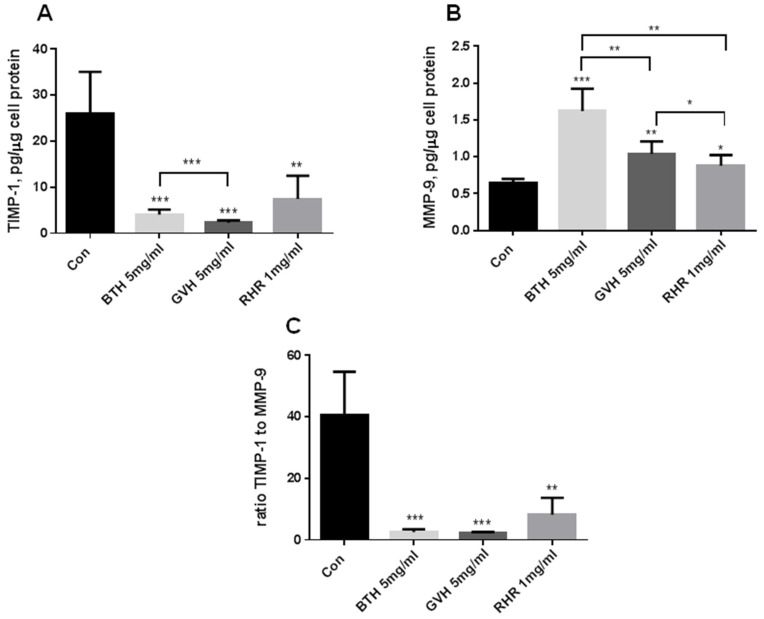
The effect of *Bidens tripartite* herb (BTH), *Galium verum* herb (GVH), and *Rumex hydrolapathum* root (RHR) on the endothelial cell production of angiogenic MMP-9 (**A**), and angiostatic TIMP-1 (**B**) released in the culture medium. (**C**) represents the relationship between MMP-9 and TIMP-1. Endothelial cells were exposed for 24 h to the control medium, and medium supplemented with herbal extracts at the highest concentration that does not change cell viability. The data were interpreted with repeated measures analysis of variance–One-way ANOVA, using a post hoc test for multiple comparisons (Dunn’s or Tukey’s tests). The results are expressed as mean ± SD, derived from three independent experiments, and were calculated per µg of cell protein. Abbreviations: TIMP-1- tissue metallopeptidase inhibitor 1, MMP-9–metalloproteinase 9 (collagenase type IV or gelatinase B), ratio TIMP-1/MMP-9–the relationship between TIMP-1 and MMP-9. Statistical significance: * *p* < 0.05, ** *p* < 0.01, *** *p* < 0.001.

The appropriate ratio between metalloproteinases and their inhibitors is essential for angiogenesis. All herbal extracts reduce the production of TIMP-1 (Figure 4A), which resulted in increased MMP-9 concentrations (Figure 4B). This effect reflects the low ratio between TIMP-1 and MMP-9 (Figure 4C), and favor pro-angiogenic invasiveness (Figure 2C).

## 3. Discussion

Angiogenesis depends on endothelial proliferation, migration, and invasiveness., The teamwork of endothelium, ECM, and angiogenic/angiostatic factors provide normal vessel sprouting [1,2]. Angiogenesis is also a fundamental step in tumorigenesis. Compounds with potentially antiangiogenic effects are tested using cancer cells to detect their impact on cell proliferation. In our study, we demonstrated how BTH, GVH and RHR can influence angiogenesis. To this end, we tested proliferation, migration, and invasiveness crucial for new blood vessels formation. Furthermore, we detected the production of angiogenic, angiostatic factors, anti-inflammatory, and antioxidative properties. Among the three tested herbal ethanolic extracts, BTH seems to have the most significant potential to modify the angiogenesis process. BTH significantly reduces cell proliferation, migration, and increases cell invasiveness when compared with the control group. Summarizing the results of BTH, it can be concluded that anti-proliferative, anti-migrative, and a relatively low impact on endothelial invasiveness allow BTH to be classified as an antiangiogenic extract.

The previous data showed that *B. tripartite* inhibited the proliferation of different cancer cell lines (cervical, liver, pancreatic, breast) in vivo and in vitro. This activity can result from the main extracts’ compounds, such as isoquercetin [28,29] and chlorogenic acid [6]. Also, other active compounds from *Bidens* species, such 1,2-dihydroxy-5(E)-tridecene-7,9,11-triyne, 1,2-dihydroxytrideca-5,7,9,11-tetrayne, and1,3-dihydroxy-6(E)-tetradecane-8,10,12-triyne inhibited the HUVECs proliferation and migration [30]. These data are consistent with our results which indicate the high activity of BTH extract.

The angiogenesis is also affected by GVH and RHR extracts, but this effect is less pronounced. Schmidt et al. documented that the aqueous extract of *G. verum* suppressed the growth and invasion of various laryngeal, head, and neck cancer cell lines [9]. At the same time, Yagasaki et al. confirmed the association between the inhibition of invasion in rat teratoma cell lines and the presence of chlorogenic acid in *G. verum* herb [31]. Our data do not confirm previous reports. So far, no research has studied the GVH’s ability to inhibit endothelial proliferation and migration. Similarly to BTH, GVH reduces cell proliferation but does not change cell migration, decreasing RHR. As demonstrated by Shiwani et al. and Later et al., Rumex species have diverse abilities to slow down the cell cycle of many cancer cells (colon, squamous, breast) [23,32]. According to the Lee et al. study, the antiproliferative activity may be associated with anthraquinone, emodin strongly interfering with the proliferation of different tumor cell lines [33]. The above-presented findings show the influence of RHR on cell proliferation, also confirmed in our study of the RHR ethanolic extract. Additionally, we also demonstrated that RHR ethanolic extract decreases HUVEC cell migration. Our study revealed, for the first time, the antiangiogenic activity of GVH and RHR by influencing the proliferation and migration of HUVEC cells, except for a poorly marked effect of GVH on cell migration.

The most potent proangiogenic factor for endothelial cells is the vascular endothelial growth factor (VEGF). It stimulates endothelial cell proliferation and migration in response to tissue hypoxia [34]. The constitutive production of VEGF in HUVECs in a standard culture condition (normal oxygen pressure) is meager and under the detection limit of most commercially available kits [35]. Pro-angiogenic factors (PDGF- platelet-derived growth factor–PDGF, and HGF–hepatocyte growth factor, hepatocyte growth factor–HGF), including pro-inflammatory cytokines (IL-6, IL-8), are reduced by herbal extracts. The examples of medicinal plants or phytocompounds known to have anti-proliferative and anti-migratory activity in PDGF and HGF-induced endothelial cells are: *Salviae miltiorrhizae radix*, *Puerariae lobatae radix* [36], and saponins from the root of *Pulsatilla koreana* [37]. Therefore, in our studies, we evaluated the influence of BTH, GVH, and RHR ethanolic extracts on the factors affecting the process of angiogenesis, such as PDGH, HGF, IL-6, IL-8, MMP-9(matrix metalloproteinase 9), and TIMP-1 (tissue inhibitor of matrix metalloproteinase 1).

The measured effect is particularly marked in BTH and GVH, but the BTH effect on PDGF and HGF is less pronounced. Similarly, RHR’s anti-inflammatory effect is weaker than BTH and GVH. RHR only reduced the production of the chemokine IL-8 and did not change IL-6 production. The balance between MMPs and TIMPs favors normal angiogenesis. All herbal extracts reduce the production of TIMP-1, with concomitantly increased MMP-9 concentrations. It reflects the low ratio between TIMP-1 and MMP-9 and favors pro-angiogenic invasiveness.

Many pro-inflammatory cytokines contribute to the angiogenesis process. IL-6 augments VEGF production [38] and the permeability of forming vasculature characteristic of many disorders [39]. IL-8, in autocrine and paracrine signaling, stimulates endothelial cell proliferation, extracellular matrix degradation, and capillary formation [40]. BTH and GVH ethanolic extracts vigorously reduce pro-inflammatory IL-6 and IL-8 cytokines compared to the control cells. RHR effect is less potent than BTH and GVH. It only reduced the production of the CXCL8 (IL-8) chemokine and did not change IL-6 production. Scientific data indicate that the inhibitory effect of different *Bidens* species (*B. bipinnata, B. pilosa*) and *G. verum* on pro-inflammatory cytokines (IL-6, IL-1β, TNF-α, IL-8) are documented using various cells such as macrophages, keratinocytes, and also HUVECs [12,41]. It is also known as chrysophanol, the active compound of *R. crispus,* and physcion 8-*O*-β-glucopyranoside isolated from *R. japonicus,* significantly suppressed the IL-6 level in vitro [19,42,43], and in vivo [24,42]. It can be assumed that the extract may impact IL-6 secretion depending on cell types. The in vitro experiments were carried out using various cells: mouse splenocytes, human mast cells, monocyte/macrophage cells, and mesangial cells but not HUVECs, where we did not observe the Il-6 inhibitory effect.

The appropriate ratio between metalloproteinases and their inhibitors is essential for angiogenesis. All tested herbal extracts (BTH, GVH, and RHR ethanolic extracts) reduce the production of TIMP-1, which results in increased MMP-9 concentrations. This effect reflects the low ratio between TIMP-1 and MMP-9 and favors pro-angiogenic invasiveness. The results of earlier work indicate that *B. Pilosa* L. decreased MMP-8 (neutrophil collagenase) and TIMP-1 production in rats [44]. Schmidt et al. showed different gelatinolytic activity (MMP-2, MMP-9) and TIMPs concentrations in various cancer cells [9]. However, according to reports of Jin et al., one of the main compounds, chlorogenic acid occurring in both extracts of *G. verum* and *B. tripartita,* may be considered a potent suppressor of MMP-9 activity [45] and responsible for the antiangiogenic activity of the herbs. Similarly, *Rumex species* also has an antiangiogenic nature. Physcion 8-*O*-β-glucopyranoside, isolated from *R. japonicus*, decreased MMP-2 and increased TIMP-3 production in ovarian cancer cells [46]. In contrast, anthraquinones reduced the production of ECM proteins–collagen IV and fibronectin–substrates for MMPs [47]. Our finding suggests that the extracts from these plants favor the pro-angiogenic invasiveness of HUVEC due to the MMP-9 increase and TIMP-1 decrease. Furthermore, the rise of MMPs is associated with the release of growth factors such as VEGF, affecting the HUVEC cell proliferation, migration, and differentiation [48].

Angiogenesis and inflammation are associated with generating reactive oxygen species (ROS). ROS created within the cells initiates oxidative stress-induced angiogenesis. Among the three herbal extracts, RHR ethanolic extract exerted the highest antioxidant activity, and its antioxidant activity was evaluated for the first time. However, extracts of different *Rumex* species (flowers, seeds, leaves, roots, stems) have already been studied in this respect. The radical scavenging capacity of *R. patientia* [49,50], *R. crispus* [21,22,23], and *R. obtusifolius* [21] has been previously demonstrated. In addition, the different studies suggest the antioxidant potency of *Rhumex* sp. extracts can result in chrysophanol and physcion presence [22]. The GVH and BTH extracts were also characterized by antioxidant activity, however less than the RHR extract. The antioxidant potential of the methanolic extract of *G. verum* was proven by other authors using different in vitro models (nitric oxide radical scavenging assay, neutralizing hydrogen peroxide test, and reducing OH radical formation) [13,14]. Similarly, the antioxidant action of methanolic and ethanolic extracts from *B. tripartita*, using DPPH, ABTS, CUPRAC, FRAP, and phosphomolybdenum assays, were also demonstrated in the different studies [5,6,7]. The studies of *B. tripartita* indicate that activity results from flavonoids [7] and phenolic acids [5,6,7].

In addition, the high content of polyphenols in RHR extract was detected in our study. The assessed amount was comparable to previous data performed with *R. patientia* (315 mg GAE/g of extract) [50] and *R. crispus* (211.2 mg GAE/g) [23]. In turn, our research, for the first time, determined the content of polyphenols in the ethanol extract of GHR. Results of previous trials of *G. verum* indicated significant differences in the content of these compounds in methanolic extracts ranging from 2.44 mg/g to 753 mg/g [13,14]. The observed differences could be due to differences in the preparation of the extract or the characteristics of the raw material for testing [13,14]. Ethanolic and methanolic extracts from BTH tested by Orhan et al. and Uysal et al. are characterized by more polyphenols than our BTH extract results [5,6].

Our results proved the correlation between the antioxidant activity of extracts and their total polyphenolic content. The literature data presents that antioxidant activity and the content of catechins, phenolic, and proanthocyanidins in *R. crispus* and *R. obtusifolius* have been correlated [21]. In prior studies, the antioxidant activity of *B. tripartita* resulted from the high content of polyphenols and flavonoids was also confirmed. This dependence is also observed for different plant extracts and their antioxidant activity [51].

## 4. Materials and Methods

### 4.1. Chemicals

Ethanol, methanol, sodium carbonate, sodium acetate-3-hydrate, acetic acid, and dimethyl sulfoxide (DMSO) were purchased from Avantor Performance Materials Poland S.A. (Gliwice, Poland); 2,2′-diphenyl-2-picrylhydrazyl radical (DPPH), Folin-Ciocalteu’s phenol reagent, ascorbic acid, gallic acid, 2,4,6-tris(2-pyridyl)-s-triazine (TPTZ), hydrochloric acid and FeCl_3_·6H_2_O from Sigma-Aldrich (Saint-Louis, MO, USA).

### 4.2. Plant Material

*B. tripartite herb* (BTH), *G. verum* herb (GVH), and *R. hydrolapathum* root (RHR) were purchased from Polish producer of medicinal herbs the Department of Herbal Packaging, FLOS Poland. The voucher specimens (No BH-2017, GVH-2017, RR-2017) were deposited at the Department of Pharmacognosy, Poznan University of Medical Sciences, Poland.

### 4.3. Extracts Preparation

Five grams of BTH, GVH, and RHR were extracted in 96% ethanol three times, each at 100 mL (solid-liquid ratio 1:20 for separated extraction), for 30 min at 50 °C on an ultra-sound bath. The extracts were combined and then concentrated under a vacuum at 40–50 °C until they dried. The residues were diluted with 5.0 mL of DMSO to yield stock solutions (1.0 g dry herb/1.0 mL) that were used in further experiments. Final concentrations of the BTH, GVH, and RHR extracts are expressed as mg of dry herb/plant material per mL.

### 4.4. GC-MS Analysis of the Extracts

The separation and identification of extracts’ components were achieved using a GC-MS chromatograph (SCION TQ, BRUKER). The concentrated alcohol extract was filtered through 0.2 µm and injected (1 µL) onto the column. The chromatograph was equipped with a VF-5ms Crawford Scientific silica column (30 m × 0.25 mm × 0.39), df = 0.25. The electron energy was 70 eV, and the ion source was at 200 °C. Helium was used as the carrier gas at a flow rate 1.0 mL/min. Temperature program: Enable Coolant at 50.0 °C, Coolant Timeout 20.00 min, Stabilization Time 0.50 min.; Temperature 60.0 °C, Hold 3.00 min., Total 3.00 min.; Temperature 280.0 °C, Rate 10.0 °C/min., Hold 35.00 min., Total 60.00 min. The compounds’ identification was based on comparing their retention time and mass spectra with those stored in the NIST library.

### 4.5. Antioxidant Activity and Total Polyphenolic Content

#### 4.5.1. 2,2-diphenyl-1-picryl-hydrazyl-hydrate (DPPH) Analysis

The DPPH assay was conducted according to Kikowska et al. [52] with modifications. Briefly, 25 μL of previously prepared dilutions were applied to the plate extracts and 175 μL of DPPH solution (0.2 mM solution of DPPH• radical in methanol). The plate was incubated at room temperature for 30 min. The absorbance was measured at λ = 517 nm (blank contained 25 μL of DMSO and 175 μL of methanol). Analyses were performed in six replicates. Vitamin C as a standard was used at the following concentrations: 15–120 µg/mL with R^2^ = 0.9978. The results were expressed as the IC_50_. IC_50_ values were calculated from the plotted graph of the scavenging activity of DPPH (%) against the final concentrations (in the well) of the extract/standard.

#### 4.5.2. The Ferric Reducing Antioxidant Power (FRAP) Analysis

The FRAP assay was performed according to Tiveron et al. [53]. Briefly, 25 μL of previously prepared dilutions were applied to the plate extracts and 175 μL of FRAP solution (the FRAP mixture contains 25 mL of the acetate buffer pH = 3.6 and 2.5 mL of 10 mM TPTZ in 40 mM HCl with 2.5 mL of 20 mM FeCl_3_ 6H_2_O aqueous solution). The plate was incubated at 37 °C for 30 min. The absorbance was measured at λ = 593 nm (blank contained 25 μL of DMSO and 175 μL of FRAP mixture). The standard curve was linear in the 20–80 μg vitamin C/mL range with R^2^ = 0.9993. The results were expressed as the IC_0.5_ (μg/mL), corresponding to the final extract concentration (in the well) required to produce a 0.5 O.D. value.

#### 4.5.3. Determination of Total Phenolics Content (TPC)

TPC in the ethanol extracts was determined by using the Folin-Ciocalteu reagent with slight modification [54]. Briefly, 200 µL of distilled water was added sequentially to each well, 25 µL of test extract/reference, 15 µL of Folin-Ciocalteu reagent, and 60 µL of 20% sodium carbonate solution (blank contained 200 µL of distilled water, 25 µL of DMSO, 15 µL of Folin-Ciocalteu reagent, 60 µL of 20% sodium carbonate solution). The plate was incubated in the dark at room temperature for 30 min. The absorbance was measured at λ = 760 nm. TPC in the extracts was expressed as mg of gallic acid equivalent per g of the dry herbs (plant material), utilizing a calibration curve of gallic acid (y = 0.0823x + 0.0033; R^2^ = 0.9997) in a concentration range of 2–64 μg/mL.

### 4.6. Bioactivity Assay—In Vitro Experiments

#### 4.6.1. Cell Culture

Experiments were performed using immortalized human umbilical vein endothelial cells HUVECs line EA.hy926 (kindly provided by Dr. CJ Edgell, University of North Carolina, Chapel Hill, NC, USA) [55]. The cells were routinely maintained in the Earl’s-buffered M199 culture medium, supplemented with amphotericin (2.5 μg/mL), gentamycin (50 μg/mL), L-glutamine (2 mmol/L), hydrocortisone (0.4 μg/mL), and 10% *v*/*v* fetal calf serum (Invitrogen, Waltham, MA, USA). The medium was also supplemented with a small dose of ciprofloxacin (0.5 μg/mL) as protection against *Mycoplasma* intoxication. The cells were cultured in a humid atmosphere with 5% carbon dioxide at 37 °C.

To ensure that BTH, GVH, and RHRextracts were not contaminated with endotoxin, extracts were tested with the LAL assay (Thermo Fisher Scientific, Rockford, IL, USA). The sample was considered as not contaminated when the endotoxin level was less than 0.01 ng/mL. It was considered acceptable and was involved in further in vitro studies.

Unless indicated otherwise, all of the reagents were purchased from Sigma-Aldrich (St. Louis, MO, USA). Cell culture plastics came from Nunc (Roskilde, Denmark) and Costar (Glendale, AZ, USA).

#### 4.6.2. Experimental Design

At the beginning of the research, endothelial cells were exposed to the culture medium supplemented with increasing concentration (0.5–5.0 mg/mL) of BTH, GVH, and RHR extracts to detect cell viability. This test allows us to choose the highest concentration that does not change cell viability. The following concentrations of the herb extracts were selected for further research: BTH–5 mg/mL; GVH–5 mg/mL; RHR–1 mg/mL.

In all experiments which test proliferation, migration, invasiveness and the production of angiogenic/angiostatic mediators, endothelial cells were exposed for 24 h to the control medium and medium supplemented with herbal extracts at the highest concentration that does not change cell viability.

All experiments were performed in a humid atmosphere with 5% carbon dioxide at 37 °C.

#### 4.6.3. Cell Viability

The viability of endothelial cells after exposure to a medium supplemented with herbal extracts was determined using the Trypan blue exclusion test. After the experiments, HUVECs were harvested using trypsin solution, suspended in Hanks solution and mixed in a ratio of 1:1(*v*/*v*) with 0.4% Trypan blue solution. After 10 min, the number of blue-stained nonviable cells was counted in a hemocytometer. The data were expressed as a percent of viable cells.

#### 4.6.4. Proliferation Assay

Cell proliferation was measured using the MTT assay, which measures the metabolic conversion of the MTT salt (3-[4,5-dimethylthiazol-2-yl]-2,5-diphenyl-tetrazolinum bromide) by active dehydrogenases [56]. The test was performed as described previously [57]. Briefly, after 24 h exposure to herbal extracts, the cells were incubated in a medium containing 1.25 mg/mL of the MTT salt for 4 h at 37 °C. The formazan product was dissolved with an acidic solution of 20% *w*/*v* sodium dodecyl sulfate and 50% *v*/*v* N, N-dimethylformamide. The absorbance of the converted dye was recorded at 595 nm. The data were expressed as a percentage of the control group (cells cultured in a standard medium).

#### 4.6.5. Migration/Invasion Assay

The endothelial migration was assessed using a 96-well Cell Migration Chamber (Boyden chamber) with 8 μm pore-size membranes (Cultrex, Glendale, AZ, USA; R&D Systems, Minneapolis, MN, USA). The cells at ~70% confluence were incubated in a culture medium supplemented with herbal extracts for 24 h. a humid atmosphere with 5% carbon dioxide at 37 °C. Next, the cells were harvested, washed, resuspended in a serum-free medium (SFM), and placed in an upper migration chamber previously coated with a coating buffer (for the migration assay) and a basement membranes extract (for the invasion assay), at a density of 20.000 cells per 50 µL SFM. The cells were then stimulated with the standard medium ± herbal extracts for 24 h at 37 °C. The migrated cells were detached, lysed, and labelled with a calcein AM according to the manufacturer’s instructions. Sample fluorescence was measured with a fluorescence microplate reader (Perkin Elmer, Waltham, MA, USA) using 480 nm and 520 nm wavelengths for excitation and emission, respectively. The data were expressed as a percentage of the control group (cells cultured in a standard medium).

#### 4.6.6. Cytokine Measurements

Endothelial cells were cultured with medium supplemented with ± herbal extracts for 24 h in a humid atmosphere with 5% carbon dioxide at 37 °C. The media were collected and analyzed for the constitutive concentrations of angiogenic/angiostatic mediators: PDGF, HGF, IL-8, IL-6, TIMP-1, and MMP-9. According to the manufacturer’s instructions, the mediators were measured using DuoSet Immunoassay Development Kits (R&D Systems). The assay’s sensitivity was: 28.1 pg/mL for the PDGF, 32.6 pg/mL for the HGF, 4.45 pg/mL for IL-8, 2.6 pg/mL for the IL-6, 16.9 pg/mL for the TIMP-1, and 42.4 pg/mL for the MMP-9. The results were normalized per cell protein in the culture wells. The protein concentration was measured using the Bradford method.

### 4.7. Statistical Analysis

Statistical analysis was performed with analysis of variance and post-hoc test using GraphPad Prism^TM^ 6.00 software (Graph Pad Software Inc., San Diego, CA, USA). Results were expressed as means ± SD. A *p*-value of less than 0.05 was considered significant. The IC_50_ values were calculated with Prism^TM^ 6.00 software using nonlinear regression.

## 5. Conclusions

I. BTH, GVH, and RHR can modify angiogenesis at various levels. BTH has the most significant antiangiogenic properties.
II. BTH and GVH have the most potent anti-inflammatory properties.
III. The RHR has the highest antioxidant activity. Its antioxidant potency correlated with the polyphenols content.
IV. The modifying influence of examined extracts can be promising in disorders with pathogenesis related to free radicals formation, inflammation, and angiogenesis. BTH is the best choice among the three tested extracts with its antiangiogenic and anti-inflammatory properties.

## Figures and Tables

**Table 1:** Compounds identified in the alcoholic extracts of *Bidens tripartite* herb (BTH), *Galium verum* herb (GVH), and *Rumex hydrolapathum* root (RHR) using GC-MS.

Plants, Extract	Rt (Min.)	Compounds	% Of Total	Formula
BTH extract	11.512	1-tert-butyl-3-(1-methylcyclohexyl)-2-aziridinone	13.71	C_13_H_23_NO
	12.238	ascaridole epoxide	1.13	C_10_H_16_O_3_
	17.318	1,2,3,4-tetrahydro-3-O-methyl-papaveroline	22.14	C_17_H_19_NO_4_
	18.190	3,7,11,15-tetramethyl-2-hexadecen-1-ol	4.26	C_20_H_40_O
	18.236	(1a,2ß,4ß)- 4-(1,1-dimethylethyl)-dimethyl ester 1,2-cyclopentanedicarboxylic acid	0.27	C_13_H_22_O_4_
	18.253	3-hydroxy-dodecanoic acid	0.36	C_12_H_24_O_3_
	18.272	(3ß,5a)-2-methylene-cholestan-3-ol	0.45	C_28_H_48_O
	18.442	(Z)-2-(9-octadecenyloxy)-ethanol	0.73	C_20_H_40_O_2_
	18.784	hanphyllin	5.99	C_15_H_20_O_3_
	18.922	santonin	1.34	C_15_H_18_O_3_
	19.510	n-hexadecanoic acid (syn. palmitic acid)	17.99	C_16_H_32_O_2_
	19.906	(Z,Z)-9,12-octadecadienoic acid	29.60	C_18_H_32_O_2_
	22.918	2-[4-methyl-6-(2,6,6-trimethylcyclohex-1-enyl)hexa-1,3,5-trienyl]cyclohex-1-en-1-carboxaldehyde	2.02	C_23_H_32_O
Total			100.00	
GVH extract	11.696	7-ethyl-4-decen-6-one	45.95	C_12_H_22_O
	12.226	ascaridole epoxide	4.69	C_10_H_16_O_3_
	16.664	4-hydroxy-benzenepropanoic acid (syn. p-hydroxyhydrocinnamic acid)	41.40	C_9_H_10_O_3_
	18.803	(E,Z,Z)-2,4,7-tridecatrienal	4.24	C_13_H_20_O
	19.499	estra-1,3,5(10)-trien-17ß-ol	3.72	C_18_H_24_O
Total			100.00	
RHR extract	8.879	2,2′,6,6′-tetramethyl-4,4′-biscyclohexanone	2.27	C_16_H_26_O_2_
	9.873	2-propyl-tetrahydropyran-3-ol	5.75	C_8_H_16_O_2_
	11.129	6-acetyl-ß-d-mannose	0.24	C_8_H_14_O_7_
	11.485	4-methyl-4-hepten-3-one	22.73	C_8_H_14_O
	14.792	2-myristynoyl pantetheine	21.88	C_25_H_44_N_2_O_5_S
	19.472	estra-1,3,5(10)-trien-17ß-ol	0.31	C_18_H_24_O
	19.484	oleic acid	0.41	C_18_H_34_O_2_
	19.531	androst-5-en-4-one	0.48	C_19_H_28_O
	21.088	(Z,Z,Z)- 9,12,15-octadecatrienoic acid, 2,3-dihydroxypropyl ester	0.97	C_21_H_36_O_4_
	21.163	(Z,Z)-9,12-octadecadienoic acid	3.18	C_18_H_32_O_2_
	21.366	cis-5,8,11,14,17-eicosapentaenoic acid	0.31	C_20_H_30_O_2_
	22.940	3-methyl-1,8,9-anthracenetriol	2.92	C_15_H_12_O_3_
	23.230	1,8-dihydroxy-3-methyl-9,10-anthracenedione (syn. chrysophanol)	32.04	C_15_H_10_O_4_
	25.605	5,10-dihydroxy-2-methoxy-7-methyl-1,4-anthracenedione	6.50	C_16_H_12_O_5_
Total			100.00	

**Table 2:** Total polyphenols and antioxidant activity of extracts of examined medical plants.

Examined Extract/Standard	Total Phenolic Content TPC (mg GAE/g Raw Materials)	Antioxidant Activity	
		DPPH IC_50_ (mg/mL)	FRAP IC_0.5_ (mg/mL)
*Bidens tripartita* herb (BTH)	32.70 ± 2.47	1.59	0.26
*Galium verum* herb (GVH)	58.47 ± 4.40	0.87	0.14
*Rumex hydrolapathum* root (RHR)	327.79 ± 1.76	0.07	0.02
*Vitaminum C*	Not determined	0.0077	0.0041

Abbreviations: TPC—Total phenolic content, GAE—Gallic acid equivalent, DPPH—2,2′-diphenyl-2-picrylhydrazyl test, FRAP—Ferric ion reducing antioxidant parameter.

## Data Availability

Not applicable.

## Supplementary Materials

The following supporting information can be downloaded at: https://www.mdpi.com/article/10.3390/molecules28134966/s1, Figure S1: GC-MS chromatogram of *Galium verum* herb alcoholic extract. Figure S2: GC-MS chromatogram of *Bidens tripartita* herb alcoholic extract. Figure S3: GC-MS chromatogram of *Rumex hydrolapathum* root alcoholic extract.

## Sample Availability

The data supporting reported results are available in: Department of Pharmacognosy and Biomaterials and Department of Pathophysiology from Poznan University of Medical Sciences, Poznan, Poland.
