# Supplementary material for: Antiangiogenic, Anti-Inflammatory and Antioxidant Properties of Bidens tripartite Herb, Galium verum Herb and Rumex hydrolapathum Root
Source: Molecules. 2023 Jun 24;28(13):4966. doi: 10.3390/molecules28134966 (PMC10343270; doi:10.3390/molecules28134966)
Supplement: Supplementary file 1 [file molecules-28-04966-s001.zip › molecules-2420226-supplementary.pdf]

molecules

Supplementary Materials: Antiangiogenic, Anti-inflammatory and Antioxidant properties of *Bidens tripartite* Herb, *Galium verum* Herb and *Rumex hydrolapathum* Root

Katarzyna Antoniak, Elżbieta Studzińska-Sroka, Marcin Szymański, Marlena Dudek-Makuch, Judyta Cielecka-Piontek and Katarzyna Korybalska

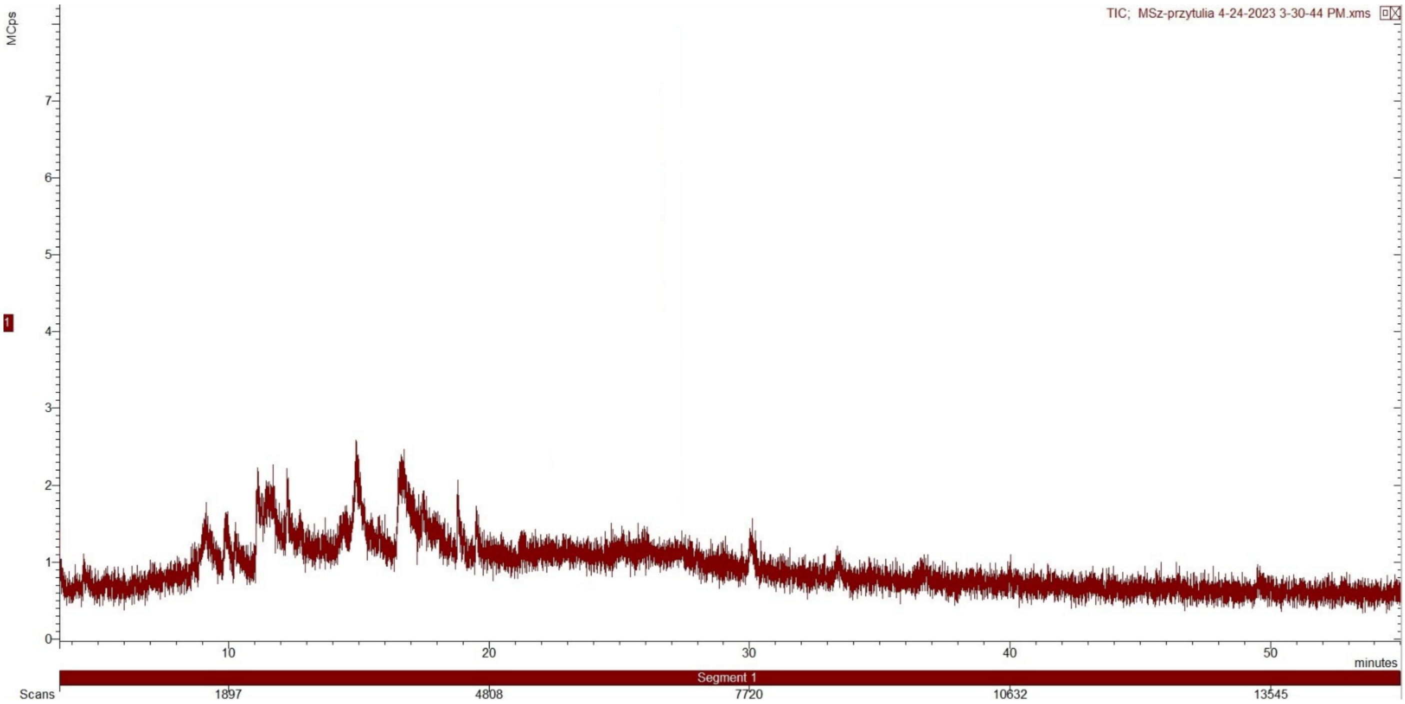

Figure S1. GC-MS chromatogram of *Galium verum* herb alcoholic extract.

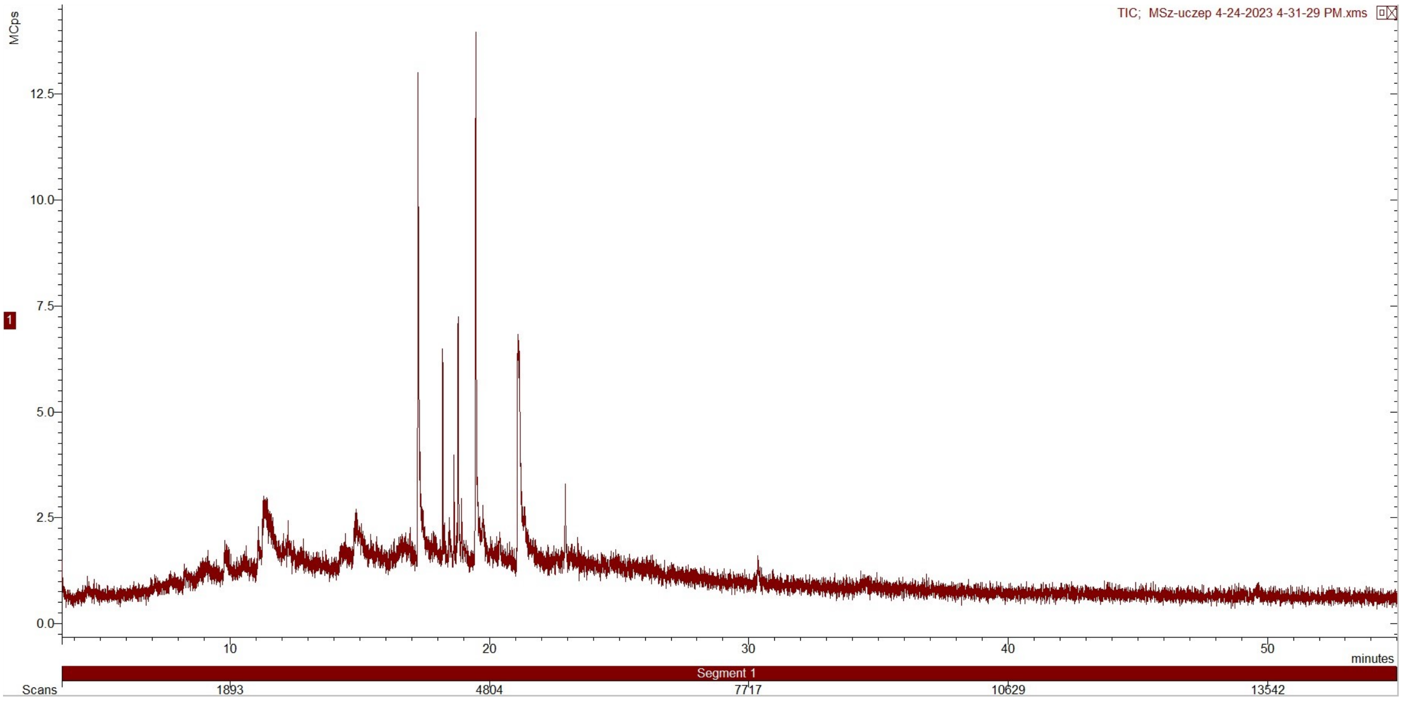

Figure S2. GC-MS chromatogram of *Bidens tripartita* herb alcoholic extract.

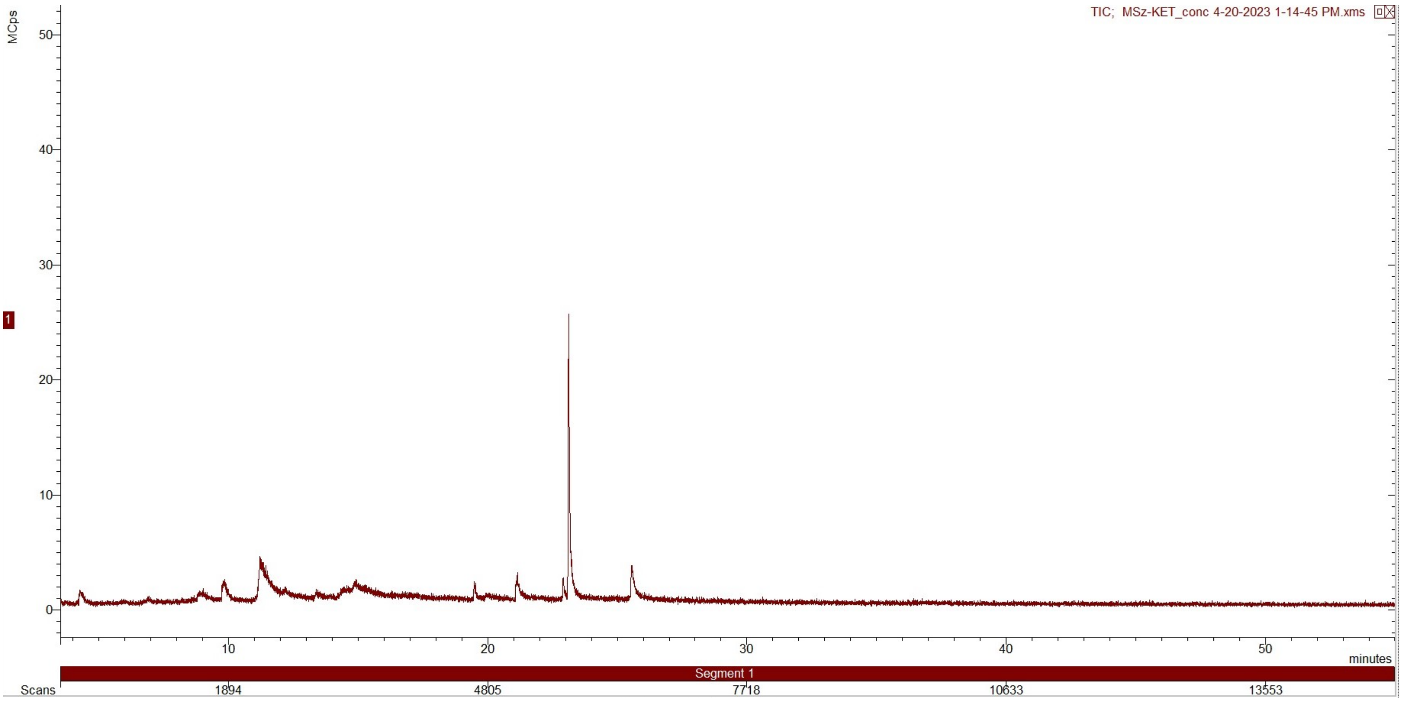

Figure S3. GC-MS chromatogram of *Rumex hydrolapathum* root alcoholic extract.
